# Recent advance of metal borohydrides for hydrogen storage

**DOI:** 10.3389/fchem.2022.945208

**Published:** 2022-08-17

**Authors:** Jianjun Liu, Yong Ma, Jinggang Yang, Lei Sun, Dongliang Guo, Peng Xiao

**Affiliations:** State Grid Jiangsu Electric Power Co, Ltd. Research Institute, Nanjing, Jiangsu, China

**Keywords:** hydrogen energy, hydrogen storage, metal borohydride, destabilization, catalysis, composite

## Abstract

Hydrogen energy is an excellent carrier for connecting various renewable energy sources and has many advantages. However, hydrogen is flammable and explosive, and its density is low and easy to escape, which brings inconvenience to the storage and transportation of hydrogen. Therefore, hydrogen storage technology has become one of the key steps in the application of hydrogen energy. Solid-state hydrogen storage method has a very high volumetric hydrogen density compared to the traditional compressed hydrogen method. The main issue of solid-state hydrogen storage method is the development of advanced hydrogen storage materials. Metal borohydrides have very high hydrogen density and have received much attention over the past two decades. However, high hydrogen sorption temperature, slow kinetics, and poor reversibility still severely restrict its practical applications. This paper mainly discusses the research progress and problems to be solved of metal borohydride hydrogen storage materials for solid-state hydrogen storage.

## Introduction

**FIGURE 1:**
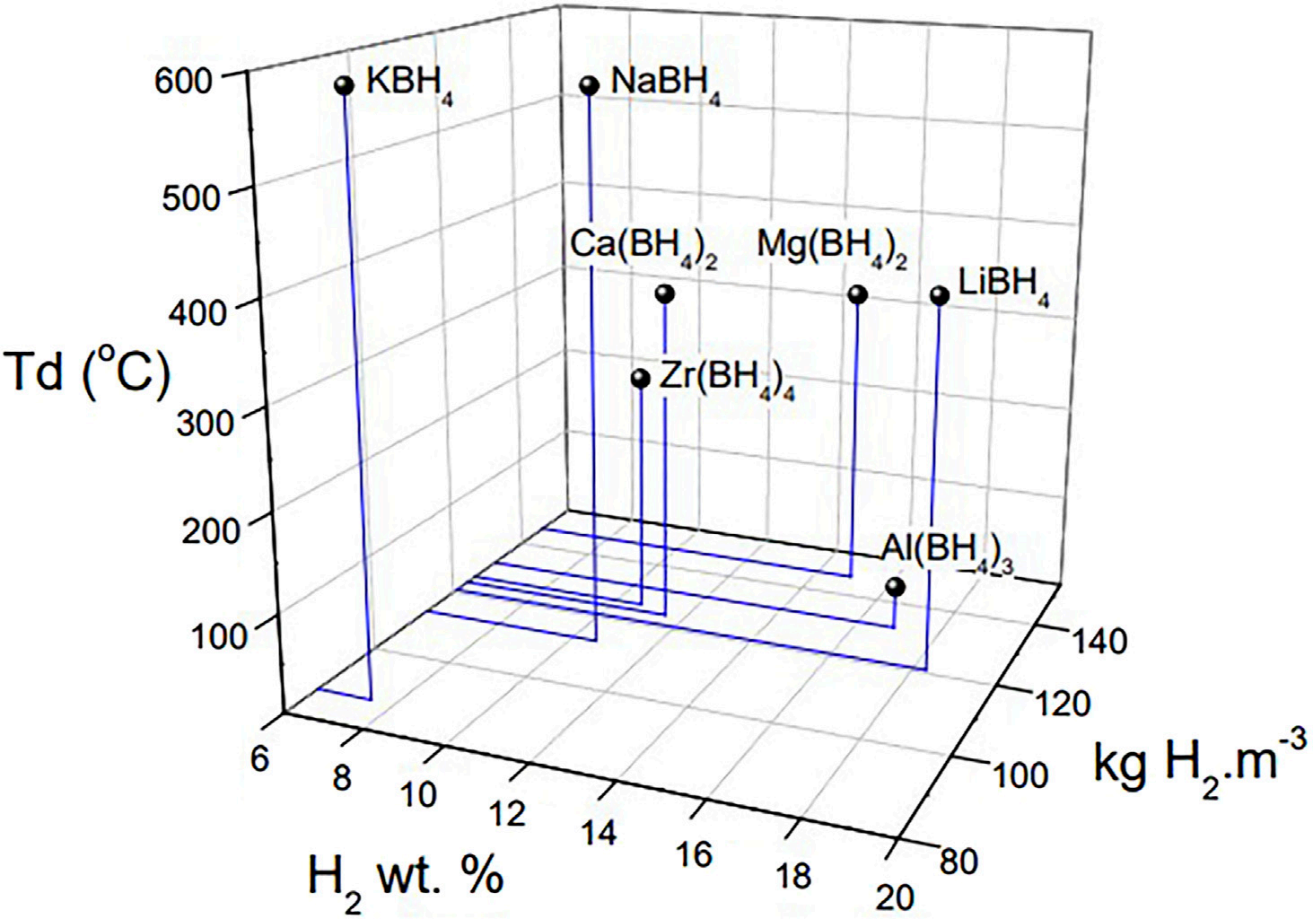
Hydrogen storage gravimetric capacity (wt.%), volume capacity (kg H_2_·m^−3^) and decomposition temperature of alkali, alkaline earth, Zr and Al borohydrides (Puszkiel et al., 2020).

Nowadays, with the shortage of fossil fuel energy and the increasingly serious environmental problems, people gradually realize the importance of finding new, efficient, environment-friendly and sustainable energy sources. In the global low-carbon energy system, hydrogen energy, as an efficient and clean new energy source, was initially used in hydrogen fuel cell vehicles fields, which then stimulated and promoted the development of hydrogen energy-related fields. The use of hydrogen energy generally includes three steps. The first is to use clean primary energy to produce hydrogen, then to store and transport the hydrogen, and finally to use the hydrogen for energy output equipment. Among them, hydrogen storage technology has become the key to the application and development of hydrogen energy due to the flammable, explosive features and low volumetric energy density of hydrogen. At present, there are three main ways of hydrogen storage: gaseous hydrogen storage, liquid hydrogen storage and solid hydrogen storage. Among them, solid-state hydrogen storage is a technology that stores hydrogen in solid hydride materials. As for the solid-state hydrogen storage mechanism, physical storage and chemical storage can be considered. The physical one is a method in which hydrogen is combined with the material in a molecular state, and hydrogen molecules are adsorbed on the surface of the material, while for the chemical one, hydrogen storage is based on a chemical absorption mechanism. In this chemical hydrogen storage material, hydrogen is combined with various elements or compounds by metal bonds, ionic bonds, or covalent bonds to form metal hydrides, coordination hydrides or chemical hydrides to achieve solid-state storage. High-capacity hydrogen storage materials composed of light elements include light metal hydrides (MgH_2_, AlH_3_), metal alanates [LiAlH_4_, NaAlH_4_, Mg(AlH_4_)_2_, etc.] metal borohydrides [LiBH_4_, NaBH_4_, Mg(BH_4_)_2_, etc.], metal nitrides [LiNH_2_, Mg(NH_2_)_2_, etc.], etc. (Schlapbach and Zuttel, 2001; Chen et al., 2002; Orimo et al., 2007; Jiang et al., 2021; Liu et al., 2021; Lu et al., 2021; Lin et al., 2022). Among these, the metal borohydrides (Figure 1) have a theoretical hydrogen storage capacity of more than 7.5 wt%, and have become a hot topic in the field of solid-state hydrogen storage research (Lv and Wu, 2021; Zhang et al., 2021; Li et al., 2022a; Wang et al., 2022a; Li et al., 2022b; Zhang et al., 2022).

However, metal borohydrides have high thermal stability, and generates highly inert elemental boron after hydrogen releasing, which affects its reverse reaction to absorb hydrogen again. Therefore, improving the reversible hydrogen storage properties of metal borohydrides has become one of the hotspots for solid-state hydrogen storage materials. This paper mainly discusses the modification methods, research progress and problems to be solved of metal borohydride hydrogen storage materials.

## LiBH_4_

LiBH_4_ is a white powder at room temperature with a melting point of about 278°C. It is insoluble in hydrocarbons, but soluble in ether, tetrahydrofuran, and liquid nitrogen. LiBH_4_ is stable at room temperature and in dry air, but it is very sensitive to moisture and protic solvents. The oxidation of LiBH_4_ with water follows Eq. 1 (Xiong et al., 2017).

$$\mathrm{LiB}H_{4}+2H_{2}O\to \mathrm{LiB}O_{2}+4H_{2}$$ (1)

LiBH_4_ has an orthorhombic structure at room temperature with a space group of *Pnma* (*a* = 7.17858 Å, *b* = 4.43686 Å, *c* = 6.80321 Å), and transforms to hexagonal system (*P*63*mc*, *a* = 4.27631 Å, *c* = 6.94844 Å) at 108−112°C (Harris and Meibohm, 2002; Soulié et al., 2002; Orimo et al., 2007; Yu and Ross, 2011). When the temperature rises to 268−286°C, LiBH_4_ begins to melt and become liquid accompanied by the liberations of 2% of the hydrogen in compound. When further heated to 380°C, LiBH_4_ begins to release a large amount of hydrogen. When the temperature reaches 600°C, LiBH_4_ can practically release a total of 9 wt% hydrogen (Zuttel et al., 2003a; Zuttel et al., 2003b). The hydrogen release reaction formula is as Eqs 2, 3:

$$\mathrm{LiB}H_{4}\to \mathrm{LiH}+B+3/2H_{2}13.8\mathrm{wt}\%$$ (2)

$$\mathrm{LiH}\to \mathrm{Li+1/2}H_{2}4.7\mathrm{wt}\%$$ (3)

The theoretical hydrogen storage capacity of LiBH_4_ is 18.5 wt%, which is higher than all hydrogen storage alloys and general coordination hydrides. However, the hydrogen contained in LiBH_4_ is not completely available, and only 13.8 wt% hydrogen is released in the range of 380°C−680°C under one bar of H_2_ pressure (Stasinevich and Egorenko, 1968; Umegaki et al., 2009). In the process of hydrogen absorption, the reversible hydrogen absorption reaction of LiH and B can be completed at 600°C and 35 MPa for 12 h (Orimo et al., 2002).

Although pure LiBH_4_ is a high-capacity hydrogen storage material, it has high hydrogen absorption and desorption temperature, slow hydrogen releasing rate, and poor reversibility. There are two common methods for modifying LiBH_4_. The first method is to thermodynamically destabilize LiBH_4_ by adding metals, metal halides, oxides, amides or metal hydrides to form composite materials or alloys after dehydrogenation (Zhang and Tian, 2011; Zhou et al., 2012; Liu et al., 2016; Zhou et al., 2017; Cheng et al., 2018; Xian et al., 2019; Ding et al., 2020; Sulaiman et al., 2021); the second method kinetically improve LiBH_4_ by using catalysts (Sulaiman et al., 2021; Zheng et al., 2021; Li et al., 2022a; Wang et al., 2022a; Li et al., 2022b) or nanoconfinement, confining LiBH_4_ in mesoporous scaffolds or mixing LiBH_4_ with nanotubes or mesoporous gels etc. (Zhou et al., 2019; Wang et al., 2020a; Le et al., 2021; Xian et al., 2021; Ye et al., 2021; Wang et al., 2022b).

### Thermodynamic destabilization

The first point that should be considered to enhance LiBH_4_ is thermodynamic destabilization. In 2006, Barkhordatian found that the 2LiBH_4_–MgH_2_ system has better hydrogen cycle thermodynamics than LiBH_4_ or MgH_2_ alone, which is believed to be because the formation of MgB_2_ “destroys” the decomposition of LiBH_4_ (Pinkerton et al., 2007).

**FIGURE 2:**
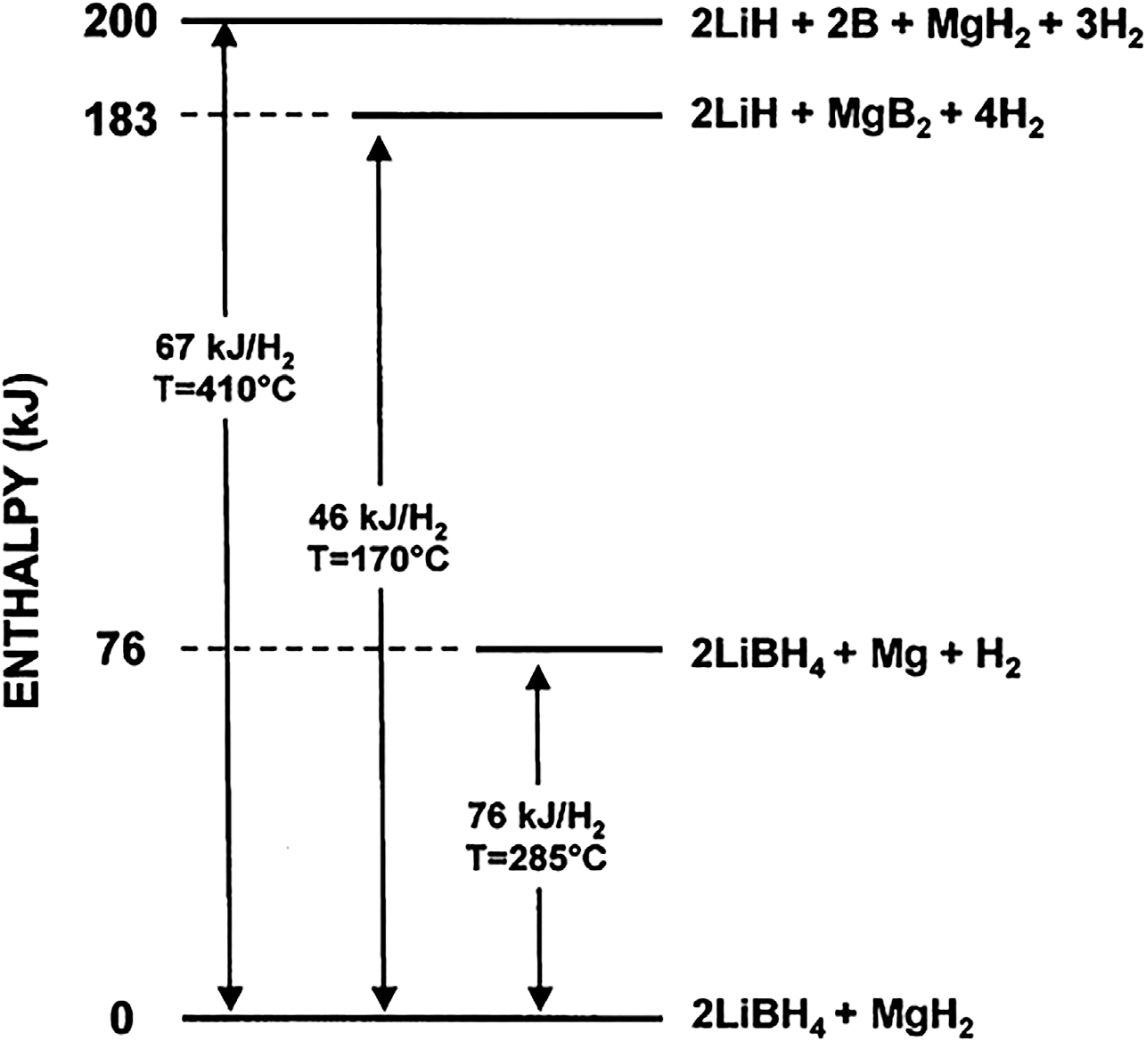
Enthalpy change of LiBH_4_-MgH_2_ after destabilization (Vajo et al., 2007).

Vajo et al. (2005) reported on the destabilization system of 2LiBH_4_–MgH_2_ and found that adding a destabilizer to LiBH_4_ to participate in hydrogen evolution can effectively reduce the reaction enthalpy change of LiBH_4_, and the enthalpy change of hydrogen evolution reaction was reduced to 46 kJ mol^−1^ H_2_ (Figure 2). Since then, researchers have carried out a lot of work around the destabilization of LiBH_4_, and have tried various destabilizing agents to improve the dehydrogenation performance and reversibility of LiBH_4_, including metal elements, metal hydrides, metal chlorides and metal oxides, etc. Among the destabilization systems, the LiBH_4_–MgH_2_ system, that is, the Li-RHC system (RHC, Reactive Hydride Complex), is highly studied. Barkohrdarian et al. patented the RHC concept in 2006. In the first published work on Li-RHC, the hydride mixture was doped with 2–3 mol% TiCl_3_ to improve the kinetic behavior, and isothermal measurements were performed in the range of 315°C–400°C, the enthalpy of hydrogen absorption down to 40.5 kJ mol^−1^. But the entropy of the Li-RHC system is different from that of the metal-hydrogen system, which is related to the [BH_4_]^–^ cluster configuration after hydrogen interaction (Vajo et al., 2005).

Bosenberg et al. (2010) published a paper, which for the first time expounded the overall hydrogenation and dehydrogenation reaction mechanism of Li-RHC under dynamic conditions. The following two-step reaction (Eq. 4) was observed during hydrogen evolution:

$$\mathrm{Mg}H_{2}+2\mathrm{LiB}H_{4}\to \mathrm{Mg}+2\mathrm{LiB}H_{4}+H_{2}\to \mathrm{Mg}B_{2}+2\mathrm{LiH}+4H_{2}$$ (4)

In the first step, MgH_2_ is desorbed and Mg is formed. After that, LiBH_4_ desorb and LiH and MgB_2_ are formed, and they found that MgB_2_–LiH composites could start to absorb hydrogen at 50 bar and 250°C, and have a much lower decomposition temperature compared to pure LiBH_4_ (Bosenberg et al., 2007). The researchers further found that the formation of B at low hydrogen pressure during dehydrogenation prevented the rehydrogenation of Li-RHC. Barkhordarian et al. (2007), Bosenberg et al. (2007) proposed that the kinetic barriers for the formation of LiBH_4_, NaBH_4_ and Ca(BH_4_)_2_ were significantly reduced when B was replaced by MgB_2_, and the kinetics of these borohydrides were enhanced. The higher reactivity of B in MgB_2_ facilitate the formation of the [BH_4_]^−^ complex. In addition, after replacing elemental B with MgB_2_, the reaction enthalpy decreased by about 10 W mol^−1^ H_2_.

The 2LiBH_4_–MgH_2_ system has different hydrogen evolution reaction paths under different hydrogen back pressures and temperatures. Only under suitable conditions (e.g., 350°C, 5.5 bar), LiBH_4_ will destabilize with MgH_2_ to form MgB_2_ and release hydrogen; otherwise, the dehydrogenation process of the system is the respective decomposition reactions of LiBH_4_ and MgH_2_ (Mao et al., 2013; Kim et al., 2015; Shao et al., 2015).

Nakagawa et al. (2007) found the effect of hydrogen back pressure on the dehydrogenation of Li-RHC, and studied the formation of MgB_2_ when dehydrogenation back pressure and inert gas were applied at 450°C, and found that only under hydrogen back pressure will MgB_2_ be formed, and the formation of MgB_2_ is considered to be a sign of a reversible reaction and is the key to the re-formation of LiBH_4_ in the reverse reaction. When heated at a back pressure below 3 bar, the LiBH_4_–MgH_2_ reaction product hardly has MgB_2_, but when the back pressure rises to 5 MPa, the product tends to be MgB_2_ (Shaw et al., 2010; Gosalawit-Utke et al., 2011; Zhou et al., 2012).

In addition to LiH–MgB_2_ composites, Li–Al–B–H is also a promising Li-RHC (Yang et al., 2007; Zhang et al., 2009; Ravnsbaek and Jensen, 2012; Ye et al., 2021). Liu et al. studied LiBH_4_–AlH_3_ composites and found that AlH_3_ can destabilize LiBH_4_. The LiBH_4_–AlH_3_ composite releases about 11.0 wt% hydrogen at 450°C for 6 h, and the kinetic performance is much better than that of pure LiBH_4_. In addition, AlH_3_ also improves the reversibility of LiBH_4_ in LiBH_4_–AlH_3_ composites, and the decomposition kinetics of LiBH_4_ is enhanced with the increase of AlH_3_ content. The 2LiBH_4_ + AlH_3_ composite can release 82% of the hydrogen capacity of LiBH_4_ within 29 min at 450°C (Liu et al., 2016; Liu et al., 2017).

Another destabilizing system that has received much attention is the Li–B–N–H composite system. The H atom in the [BH_4_]^−^ group in LiBH_4_ tends to gain electrons and is negatively charged (H^δ−^), and the H atom in NH_3_ or LiNH_2_ tends to lose electrons and is positively discharged (H^δ+^). H^δ−^ and H^δ+^ in the composite system will interact to generate hydrogen bonds, and the samples will interact during heating. It is easier to release hydrogen at low temperature than pristine LiBH_4_.

Johnson et al. (2009) found that at normal temperature and pressure, LiBH_4_ can react with NH_3_ to form LiBH_4_·NH_3_. When the temperature rises to about 40°C, NH_3_ will be released from LiBH_4_·NH_3_ and become pure LiBH_4_ instead of hydrogen. Pinkerton et al. (2005) studied the LiBH_4_–LiNH_2_ composite as hydrogen storage material for the first time. When LiBH_4_ and LiNH_2_ were subjected to high-energy ball milling at a molar ratio of 1:2 or heated to above 95°C, the mixture would *in situ* generate a new type of hydrogen storage material Li_3_BN_2_H_8_. The hydrogen storage capacity of the system is as high as 11.9 wt%, and the melting point is 190°C. The hydrogen releasing amount is 10 wt% at 250–350°C.

In a word, thermodynamic destabilization is an efficient method to tailor the hydrogen storage performances of LiBH_4_.

### Kinetic improvement

Kinetics is another point that should be considered to improve the hydrogen storage properties of LiBH_4_. Catalysis and nanoconfinements are two common methods to improve the kinetics of LiBH_4_.

It was found that the effects of many metal elements, metal oxides and halides on LiBH_4_ were both destabilizing and catalysis. Fang et al. (2008) found that the Ni/Co/Fe borides produced by the reaction between the metal element and LiBH_4_ can catalyze the reaction. A composite of LiBH_4_ and single-wall nanotubes (SWNT) can release 11.4 wt% of hydrogen within 50 min at 450°C after ball milling. Wang et al. (Kang et al., 2013; Wang et al., 2013; Wang et al., 2014a; Wang et al., 2014b; Wang et al., 2014c) studied the effect of TiF_3_ and Nb_2_O_5_ doping on the LiBH_4_–MgH_2_ system by using a three-step preparation method of mixture pre-grinding, isothermal treatment and co-grinding with MgH_2_. The study found that NbB_2_ and TiB_2_ formed during hydrogen desorption can effectively improve the performances of LiBH_4_–MgH_2_ system. The reason for improving the cycling stability of the LiBH_4_–MgH_2_ system is that the reaction products of TiF_3_ and Nb_2_O_5_ with LiBH_4_–MgH_2_ are nucleating agents, which can promote the formation of MgB_2_, thereby promoting the cycling stability.

Cai et al. (2014) studied the catalytic effect of different forms of nanostructured CoB on the hydrogen absorption and desorption process of LiBH_4_, and found that the catalytic effect of CoB is roughly in the order of mulberry-like > bayberry-like > chain-like > sheet-like > rod-like, and it is proportional to the specific surface area. At 200°C, the mulberry-like and bayberry-like CoB catalyzed LiBH_4_ have the best hydrogen evolution properties, and the hydrogen releasing amounts of the two CoB-catalyzed LiBH_4_ were 4.6 wt% and 4.8 wt%, respectively. The mulberry-like CoB exhibited the best catalytic effect at 350°C, which obtained 10.4 wt% hydrogen from LiBH_4_ with almost complete reversibility at 400°C and 10 MPa. Moreover, at the fourth cycle 9.6% of hydrogen can still be released.

Among many metal halides, TiCl_3_ and TiF_3_ have the most significant catalytic effects and have been widely studied. Adding TiCl_3_ or TiF_3_ can reduce the initial dehydrogenation temperature of LiBH_4_ to about 100°C, and TiF_3_ has a more significant catalytic effect. Ti halides react with LiBH_4_ to form Ti hydrides and Li halides when heated, and the *in-situ* formation of Ti hydrides can effectively improve the cyclic dehydrogenation performance of LiBH_4_. Ming et al. (2008) studied the LiBH_4_ + 0.2MgCl_2_ + 0.1TiCl_3_ composite catalytic system, the material can desorb 5 wt% of hydrogen at 400°C, and can absorb 4.5 wt% of hydrogen at 600°C and 7 MPa after dehydrogenation.

Zhang et al. (2017) (Li et al., 2017) studied the effect of Co(OH)_2_, Co_3_O_4_ and CoO on the catalytic performance of LiBH_4_–2LiNH_2_. It was found that the three catalysts were *in situ* reduced to form metallic Co element during the heating process, and then catalyzed the molten LiBH_4_–2LiNH_2_ sample. The hydrogen desorption temperature was effectively lowered. The initial dehydrogenation temperature of the LiBH_4_–2LiNH_2_–0.05Co(OH)_2_ sample was reduced to 70°C, which is the lowest dehydrogenation temperature reported for this system so far.

**FIGURE 3:**
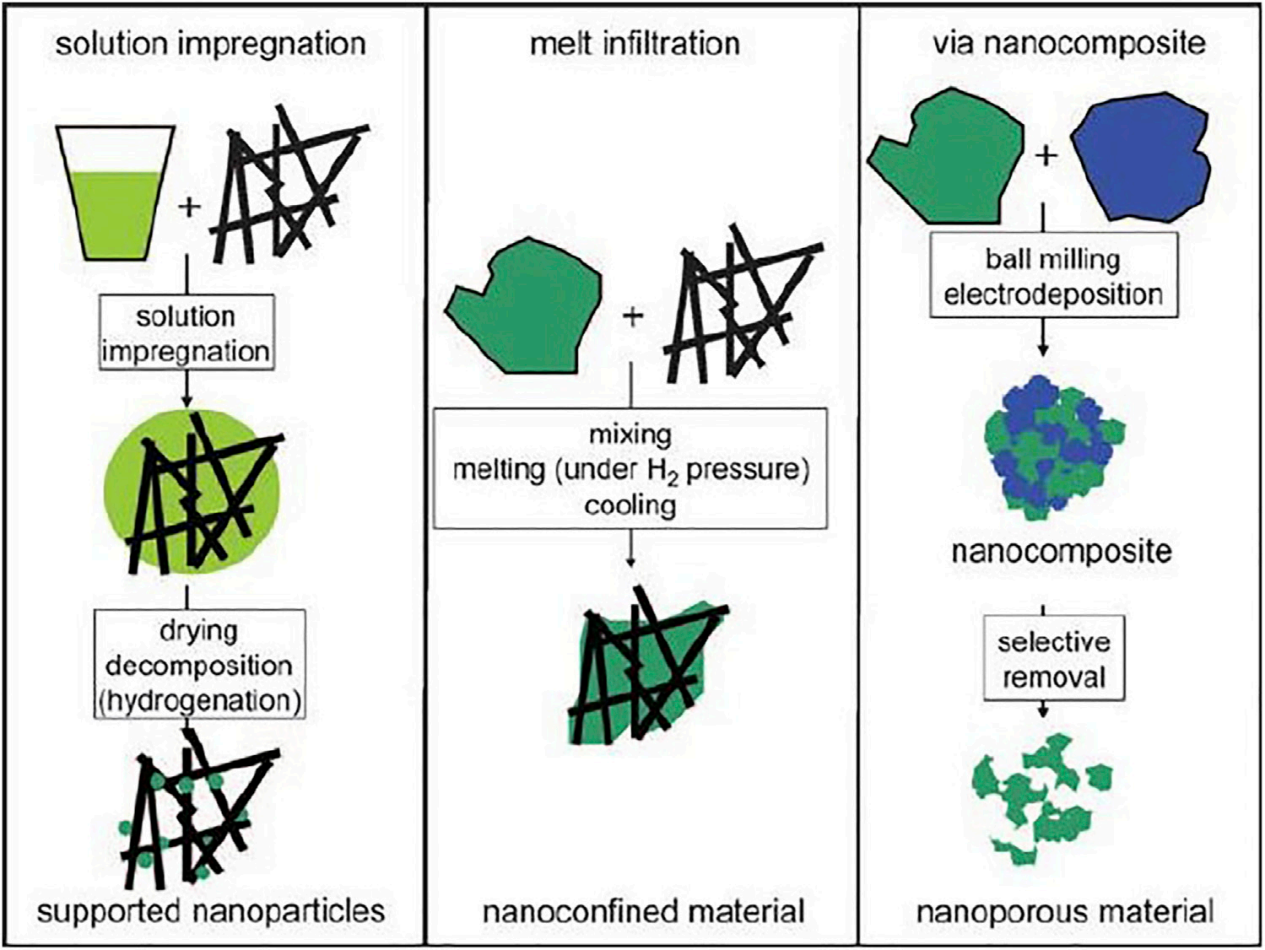
Schematic illustration of different ways to prepare nanostructured metal coordination hydrides or metal hydrides (Jongh and Adelhelm, 2010).

Nanoconfinement is another method to improve the kinetics of LiBH_4_, which is to fill the material into the nanopores, and use the interaction between the material and the nanopores to promote the reaction or limit the phase separation during sorption process. Based on this, the confinement frame material must have a high specific surface area and porosity in order to improve the loading rate and avoid the collapse of the porous material during the hydrogen absorption and desorption cycle. At the same time, the material needs to have good chemical inertness to avoid reaction with the hydrogen storage material. Materials suitable for nanoconfinement include carbon-based materials (carbon aerogel/activated carbon/ordered mesoporous carbon), metal-organic frameworks (MOFs), and mesoporous silica (SBA-15), etc. The traditional method of grain refinement, such as high-energy ball milling, gradually refines the grain through the collision between the ball and the tank. But such method is still prone to lead to the agglomeration of nanoparticles and re-agglomeration into larger particles. Different from high-energy ball milling, the method of nano-confinement is to confine the hydride particles in the pores of the frame material, which can obtain finer particles than the ball milling method, and the particles does not agglomerate during the hydrogen absorption and desorption cycle, which increases the cycling stability. At the same time, it also shortens the distance of hydrogen diffusion, and increases the number of grain boundaries, which is conducive to the progress of hydrogen absorption and desorption reactions. The schematic diagram of preparing nanostructured metal coordination hydrides or metal hydrides by ball milling, solution impregnation and melt injection methods is shown in Figure 3.

Liu et al. (2011) studied the confinement of LiBH_4_ in porous carbon with a pore size of more than 2 nm, which can effectively reduce the initial hydrogen desorption temperature of LiBH_4_ to 220°C. They further found that confining LiBH_4_ in highly ordered nanoporous carbon will lead to the disappearance of the diffraction peaks for phase transition and melting of LiBH_4_.

**FIGURE 4:**
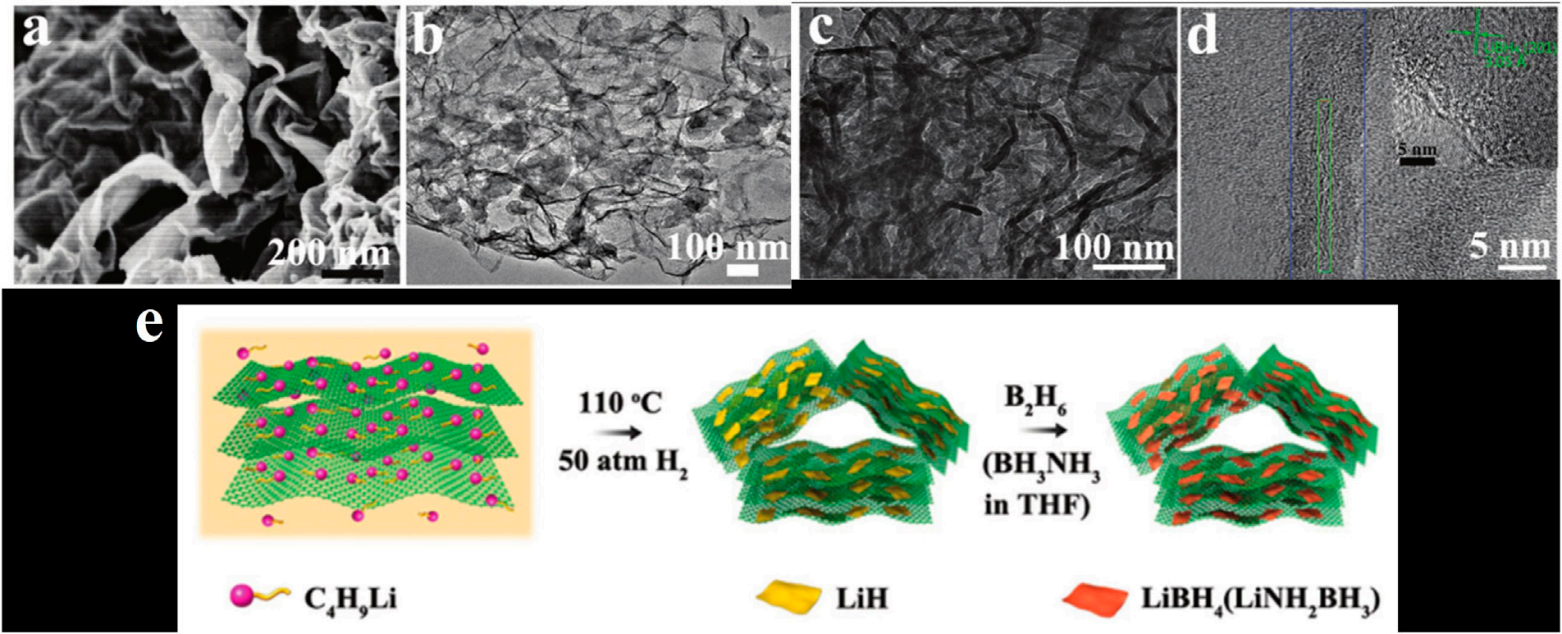
**(A)** SEM image of LiBH_4_@G. **(B–D)** TEM image of LiBH_4_@G. **(E)** Flow chart of preparation of LiH@G (Xia et al., 2017).

Xia et al. (2017) confined LiH in graphene, and then reacted LiH with B_2_H_6_ to generate nanoconfined LiBH_4_, as shown in Figure 4. The obtained material can dehydrogenate about 7.6 wt% at 280°C, and dehydrogenate about 9.7 wt.% within 60 min at 340°C. After absorbing hydrogen at 320°C and 100 bar, it still has 7.5 wt% of capacity after five cycles of hydrogen absorption/desorption.

Gross et al. (2008) confined LiBH_4_ into the pores of carbon gels, and at 300°C, the nanostructured hydrides formed by filling LiBH_4_ in the bulk of porous carbon aerogels. The measured hydrogen evolution rate was higher than that of bulk materials. The rate of hydrogen evolution is 50 times faster than the bulk materials. At the same time, it was found that compared with the bulk LiBH_4_, the hydrogen desorption activation energy of the nanoconfined LiBH_4_ was reduced from 146 kJ mol^−1^ to 103 kJ mol^−1^, and the hydrogen desorption temperature was reduced by 75°C. The nanostructured LiBH_4_ also had a better hydrogen absorption and desorption cycle performance.

Jensen (Gosalawit-Utke et al., 2011; Gosalawitutke et al., 2012) et al. melted 2LiBH_4_–MgH_2_ into carbon aerogel scaffolds and found that the performance of 2LiBH_4_–MgH_2_ has been significantly improved, and 90% of the hydrogen can be released within 90 min. The non-confined material can only release 34% of the hydrogen. Through further research, the research group melted and infiltrated 2LiBH_4_–MgH_2_–0.13TiCl_4_ in the carbon aerogel scaffold, and then tested the hydrogen absorption and desorption performance. It was found that TiCl_4_ and LiBH_4_ were successfully nanoconfined in carbon aerogel scaffolds, while MgH_2_ was only partially confined. Heated at 25°C–500°C for 5 h, the nano-confined 2LiBH_4_–MgH_2_–0.13TiCl_4_ released up to 99% of the theoretical hydrogen storage capacity, while the nano-confined 2LiBH_4_–MgH_2_ was only 94%. At the same time, the kinetics are also greatly improved. To desorb 3.6 wt% H_2_ during the first dehydrogenation, the nanoconfined 2LiBH_4_–MgH_2_–0.13TiCl_4_ takes 1.5 h, while the nanoconfined 2LiBH_4_–MgH_2_ needs 3.5 h. Moreover, the TiCl_4_ doped material possesses a dehydrogenation rate twice of the undoped material. This indicates that combination of catalysis and nanoconfinement will lead to the further improvement of the LiBH_4_-based hydrogen storage material.

Verkuijlen et al. (2012) combined the nanoconfinement of LiBH_4_ in nanoporous carbon with the addition of nickel. Nickel nanoparticles of 5–6 nm were deposited in porous carbon and then melt infiltrated with LiBH_4_. The addition of nickel has only a slight effect on the hydrogen desorption of LiBH_4_, but significantly improves the cycling performance of LiBH_4_ under mild conditions.

Although the nanoconfinement method can effectively improve the thermodynamic and kinetic properties of hydrogen storage materials, there are still many key issues to be solved, such as how to confine a large number of hydrogen storage materials into nanopores and how to achieve a high filling efficiency.

To briefly summary, LiBH_4_ possesses a very high hydrogen capacity but suffers from high thermal stability and poor reversibility. Catalized LiBH_4_-based composite such as LiBH_4_–MgH_2_ composite with proper catalysts addition can reversibly absorb and desorb hydrogen with high capacity and favored kinetics.

## NaBH_4_

NaBH_4_ is a common chemical reducing agent in the laboratory, with high thermal stability, and requires a decomposition temperature of 300°C in dry air. The theoretical hydrogen content of NaBH_4_ is 10.7 wt%, and the volumetric hydrogen storage density is 115 g L^−1^. The hydrogen desorption temperature of pure NaBH_4_ is relatively high and needs to be heated to 565°C. The hydrogen desorption reaction of NaBH_4_ is as Eq. 4. NaBH_4_ has a cubic structure at room temperature, which is the same as the crystal structure of NaCl (Urgnani et al., 2008; Liu and Li, 2009; Garroni et al., 2010; Martelli et al., 2010).

$$\mathrm{NaB}H_{4}\to \mathrm{NaH}+B+3/2H_{2}\to \mathrm{Na}+B+2H_{2}$$ (4)

At present, the methods to improve the performance of NaBH_4_ include anion and cation substitution method, destabilization method, catalysis, and particle size nanometerization. It was found that adding MgH_2_ or YF_3_ can effectively improve the thermodynamic properties of NaBH_4_. The YB6 and MgB_2_ formed during the hydrogen evolution process are more stable than the metal elements Y and B, which are the key to the reversible release of NaBH_4_ (Garroni et al., 2009).

**FIGURE 5:**
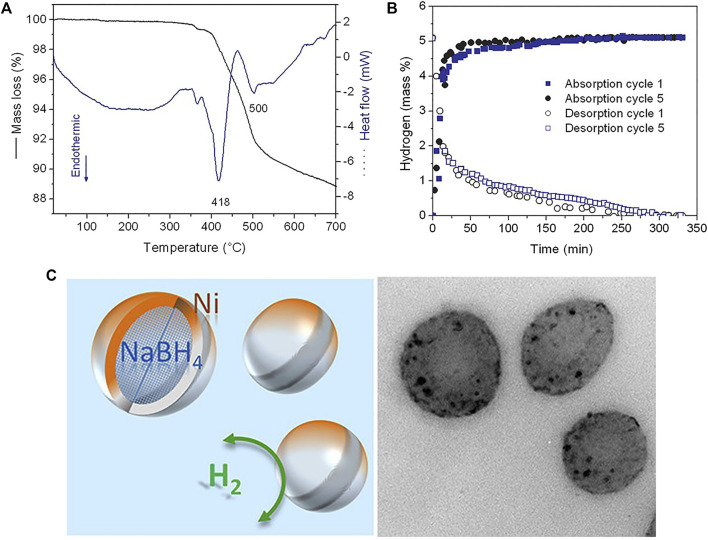
TGA/DSC curves **(A)** and isothermal hydrogen desorption curves at 350°C **(B)** of the nanoconfined NaBH_4_. **(C)** Models and TEM image of the Ni-doped nanoconfined NaBH_4_ (Christian and Aguey-Zinsou, 2012).

Ngene et al. (2011) used the liquid melting method to obtain nano-NaBH_4_ to achieve reversible hydrogen absorption and desorption performances. The initial hydrogen desorption temperature of nano-sized NaBH_4_ was reduced from 470°C of the matrix to below 250°C, and the reactant after dehydrogenation can be re-hydrogenated to NaBH_4_ under the condition of 60 bar H_2_ and 325°C. However, since the pores of this method are open, Na is volatile during the hydrogen evolution process, and only 43% of the hydrogen is stored during the cycle. Milanese et al. (2011) (Christian and Aguey-Zinsou, 2012) limited the size of NaBH_4_ particles to a few nanometers (<30 nm) by anti-solvent precipitation, which lowered its melting point and lead to hydrogen desorption beginning at 400°C. These nanoparticles can form a core after reacting with nickel chloride on their surface, which can achieve effective nanoconfinement for melting NaBH_4_ cores and their dehydrogenation products (Figure 5). Moreover, the reversibility and fast kinetics due to short diffusion lengths is also obtained.

When the catalyst is added to the hydride through ball milling, the catalyst can be uniformly distributed on the surface and grain boundaries of the hydride, which is beneficial to the dissociation and recombination of hydrogen in the hydride. It was found that adding Ti, TiH_2_, TiF_3_ are beneficial to improve the thermodynamics of NaBH_4_, reducing the hydrogen desorption temperature of NaBH_4_, and among them, TiF_3_ has the best catalytic effect. TiF_3_ and NaBH_4_ will react with each other to form TiB_2_, and this formed TiB_2_ will catalyze the decomposition of the remaining NaBH_4_, and TiB_2_ will also catalyze the regeneration of NaBH_4_ to promote the stability of the cycle (Garroni et al., 2009).

In general, the research on NaBH_4_ mainly focus on its hydrolysis or methanolysis to generate hydrogen and limited papers on its dehydriding and rehydriding were reported (Santos and Sequeira, 2011). This is partially due to the high thermal stability and rather slow kinetics. Therefore, NaBH_4_ is more suitable for hydrogen generation by a hydrolysis or methanolysis process (Saka, 2021; Saka and Balbay, 2021; Saka, 2022a; Saka, 2022b; Saka, 2022c; Saka and Balbay, 2022).

## Mg(BH_4_)_2_

The mass hydrogen storage density of Mg(BH_4_)_2_ is 14.8 wt%, and the volume hydrogen storage density is 112 g L^−1^. Mg(BH_4_)_2_ has a variety of crystal structures, and each crystal structures can transform into the other at different temperatures, which is determined by its own coordination of two [BH_4_]^–^, thus increasing the complexity of the molecular structure. There are about seven crystal structures reported for Mg(BH_4_)_2_, but although there are many crystal forms, they all transform to high-temperature stable *β* phase before hydrogen evolution, and this will lead to that different crystal forms have little effect on its hydrogen absorption and desorption properties.

The decomposition of α-Mg(BH_4_)_2_ first undergoes a phase transition at 190°C, and then decomposes into MgH_2_, Mg and MgB_2_ with the increase of temperature. The decomposition is divided into two steps (Chłopek et al., 2007; Li et al., 2007):

$$\mathrm{Mg}{\left(BH_{4}\right)}_{2}\to \mathrm{Mg}H_{2}+2B+3H_{2}\left(11.1\mathrm{wt}\%\right)$$ (5)

$$\mathrm{Mg}H_{2}+2B\to \mathrm{Mg}B_{2}+H_{2}\left(4.2\mathrm{wt}\%\right)$$ (6)

The desorption temperature of Mg(BH_4_)_2_ is lower than that of LiBH_4_, but a stable MgB_12_H_12_ is generated during the thermal decomposition of Mg(BH_4_)_2_, which makes Mg(BH_4_)_2_ have high thermodynamic stability and kinetic barrier (Yan et al., 2015). It can only be recovered under the conditions of 95 MPa and 400°C, so improving its hydrogen storage performance has become a research hotspot.

Adding transition metals and their compounds is a common method to improve hydrogen storage materials, and Ti-based compounds are one of the most commonly used additives to improve the performance of hydrogen storage materials. Bardaji et al. (2011) made TiCl_3_ and Mg(BH_4_)_2_ into nanocomposite materials, and found that the initial hydrogen desorption temperature was reduced to above 100°C, and further study shows that Mg(BH_4_)_2_ doped with NbCl_5_–TiCl_3_ nanocomposite could lead to that 5 wt% hydrogen could be released when the dehydrogenation temperature was lowered below 300°C, but the cycle performance of Mg(BH_4_)_2_ was not improved by doping.

Saldan et al. (2015) studied the effect of nickel-based additives such as nano-Ni, NiCl_2_, NiF_2_, and Ni_3_B on the hydrogen absorption and desorption performance of Mg(BH_4_)_2_. The Mg(BH_4_)_2_ was partially decomposed, and amorphous Ni_3_B appeared in the decomposition products. Ni_3_B did not participate in the reaction in the process of re-absorbing hydrogen. The addition of Ni_3_B can also effectively improve the hydrogen desorption kinetics of Mg(BH_4_)_2_, and the *in-situ* formed compound Ni_3_B is the main reason for improvement of the kinetic properties of Mg(BH_4_)_2_. Wang et al. (2020b) carried out ball milling of LiBH_4_ and MgCl_2_ to synthesize Mg(BH_4_)_2_ *in situ*. In fact, the temperature was reduced to 126.9°C, and the activation energy was reduced from 487.99 kJ mol^−1^ to 120.1 kJ mol^−1^ compared with the original Mg(BH_4_)_2_. Therefore, the hydrogen storage kinetics of Mg(BH_4_)_2_ is significantly improved. Further investigation showed that by adding NbF_5_ into the composites can also lead to better hydrogen storage properties than pristine and amorphous Mg(BH_4_)_2_. The catalyzed composite starts to release hydrogen at about 120°C with a total capacity of 10.04 wt%. The reversibility of the catalytic composite was also improved, and the catalytic composite could still release 4 wt% H_2_ in the third and fourth cycles.

Combining LiBH_4_ to form the LiBH_4_–Mg(BH_4_)_2_ composite system can also improve its hydrogen storage performance of Mg(BH_4_). Zhao-Karger et al. (2011) found that when LiBH_4_ and Mg(BH_4_)_2_ were mixed and ball-milled with molar ratio close to 1:1, the composite system would eutectic and release hydrogen at about 170°C, compared with pure Mg(BH_4_)_2_. The dehydrogenation temperature of the composite system decreased by about 100°C. Chen et al. (2012) used a Co-based catalyst to catalyze the hydrogen release of the LiBH_4_–Mg(BH_4_)_2_ system, which could make the composite system start to release hydrogen from 155°C, and at the same time, the hydrogen release rate of the system was increased by 1.6 times at 270°C.

Although researchers have tried various methods to improve the hydrogen storage performance of Mg(BH_4_)_2_, its initial hydrogen desorption temperature is still high, and the kinetic performance of Mg(BH_4_)_2_ at low temperature still needs to be improved.

## Ca(BH_4_)_2_

Ca(BH_4_)_2_ is soluble in water but does not undergo hydrolysis reaction. It can exist stably in dry air, and begins to decompose at 360°C. The theoretical mass hydrogen storage density is 11.6 wt%, while 9.6 wt% of hydrogen can practically be released.

The decomposition of Ca(BH_4_)_2_ to release hydrogen is a multi-step reaction process accompanied by the formation of various possible intermediates. The reaction equation is as Eq. 6. At 347°C–387°C, Ca(BH_4_)_2_ decomposes to form CaH_2_ and some intermediate products, and at 397–497°C, the intermediate products decompose to form amorphous B and CaB_6_. Due to the complexity of the hydrogen absorption and desorption process of Ca(BH_4_)_2_, it is quite difficult to improve its hydrogen absorption and desorption kinetics.

$$3\mathrm{Ca}(BH_{4}{)}_{2}\to 2\mathrm{Ca}H_{2}+\mathrm{Ca}B_{6}+10H_{2}$$ (6)

Kim et al. (2008) studied the effect of transition metal halides on the dehydrogenation performance of Ca(BH_4_)_2_, and found that NbF_5_ would reduce the onset hydrogen desorption temperature of Ca(BH_4_)_2_ by 20°C. Under the conditions of 350°C and 90 bar H_2_, reversible uptake of 5 wt% H_2_ can be achieved. They believe that the catalytic effect of NbF_5_ may be because its melting point is only 77°C, which is melted during the ball milling process, so that it can be better distributed in Ca(BH_4_)_2_. In addition, NbF_5_ can make Ca(BH_4_)_2_ form CaH_2−*x*_F_*x*_ solid solution phase in the process of dehydrogenation, which lead to the reverse reaction to generate CaH_2_ at 350°C and 90 bar. Minella’s further research (Bonatto Minella et al., 2011; Bonatto Minella et al., 2013; Bonatto Minella et al., 2015) found that when TiF_4_ and NbF_5_ catalysts were added, Ca(BH_4_)_2_ could generate CaB_6_ after dehydrogenation, and the formation of CaB_6_ was the key to the cyclic hydrogen absorption and desorption of Ca(BH_4_)_2_. The Ca(BH_4_)_2_ sample added with TiF_4_ and NbF_5_ catalysts can realize the reverse reaction at 350°C and 145 bar.

Chu et al. (2011) studied the composite system of Ca(BH_4_)_2_–2Mg(NH_2_)_2_ and Ca(BH_4_)_2_–2Ca(NH_2_)_2_, and found that the initial hydrogen desorption temperature of the composite system is 100°C lower than that of pure Ca(BH_4_)_2_. At 480°C, Ca(BH_4_)_2_–2Mg(NH_2_)_2_ can release 8.3 wt% hydrogen, and Ca(BH_4_)_2_–2Ca(NH_2_)_2_ can release 6.8 wt% hydrogen. Compared with pure Ca(BH_4_)_2_ samples, both have lower activation energy for dehydrogenation, and better hydrogen storage performance of Ca(BH_4_)_2_.

As a high-capacity hydrogen storage material, the hydrogen storage performance of Ca(BH_4_)_2_ can be improved by introducing catalysts and other means. But from the application point of view, there are still many issues need to be addressed for Ca(BH_4_)_2_. Many problems remain to be studied. For example, NbF_5_, which has the best catalytic effect among the catalysts, can only reduce the dehydrogenation temperature by 20°C. Although the forming composite of Ca(BH_4_)_2_ with other metal borides and amides can improve the performance of Ca(BH_4_)_2_ to a certain extent. However, the hydrogen desorption temperature of the composite system is still high, and the purity of hydrogen in the released gas is not high enough. It is still the research focus of Ca(BH_4_)_2_ hydrogen storage materials to find more effective methods to improve the hydrogen storage performance of Ca(BH_4_)_2_.

## Conclusion

The metal borohydrides commonly found as LiBH_4_, NaBH_4_, Mg(BH_4_)_2_, and Ca(BH_4_)_2_ all have a high hydrogen capacity higher than 10 wt%, which is much higher than that of the materials that have been practically applied. Borohydrides also have cyclic hydrogen absorption and desorption properties, so borohydrides are one of the main research objects of solid-state hydrogen storage materials. In this paper, the common methods of borohydride modification, such as destabilization, catalysis, nanoconfinement, etc., are summarized. These methods have improved the hydrogen storage performance of borohydrides to a certain extent, but still cannot meet the comprehensive application requirements of fast kinetics, near room temperature operation, stable hydrogen absorption and desorption cycle performance, and long cycle life. Therefore, the research on borohydrides still needs to be done to find more effective methods to improve their hydrogen storage performance.

To tailor the hydrogen storage properties of metal borohydrides, the thermodynamic destabilization and kinetic improvement should be simultaneously considered by combination of various modification methods, which is the future research direction of metal borohydrides. In addition, prototype based on some metal borohydrides should be built to verify the practical hydrogen storage performances. Although the operating temperatures of metal borohydrides are relatively high compared with the traditional hydrogen storage alloys, the practical application is still possible when combined with high-temperature solid oxide fuel cell. Therefore, metal borohydrides are still promising materials for hydrogen storage.
